# Trends and correlates of cannabis use in pregnancy: a population-based study in Ontario, Canada from 2012 to 2017

**DOI:** 10.17269/s41997-018-0148-0

**Published:** 2018-11-01

**Authors:** Daniel J. Corsi, Helen Hsu, Deborah Weiss, Deshayne B. Fell, Mark Walker

**Affiliations:** 1 OMNI Research Group, Ottawa Hospital Research Institute, Centre for Practice Changing Research, 501 Smyth Road, Box 241, Ottawa, ON K1H 8L6 Canada; 2 School of Epidemiology and Public Health, University of Ottawa, Ottawa, Canada; 3 Children’s Hospital of Eastern Ontario Research Institute, Ottawa, Canada; 4 Department of Family Medicine, University of Ottawa, Ottawa, Canada; 5 BORN Ontario, Children’s Hospital of Eastern Ontario, Ottawa, Canada; 6 Department of Obstetrics and Gynecology, University of Ottawa, Ottawa, Canada

**Keywords:** Cannabis, Marijuana, Epidemiology, Canada, Pregnancy, Women, Time trends, Cannabis, Marijuana, Épidémiologie, Canada, Grossesse, Femmes, Tendances temporelles

## Abstract

**Objective:**

Forthcoming legislative changes will legalize and make cannabis widely available in Canada. We conducted an analysis of Ontario’s birth registry to determine recent trends and correlates of cannabis use in pregnancy.

**Methods:**

We conducted a population-based retrospective cohort study assembled from the Better Outcomes Registry & Network (BORN) Ontario database, covering live births and stillbirths in Ontario between April 2012 and December 2017. Trends in self-reported cannabis use in pregnancy were analyzed according to maternal age and area-level socio-economic status (SES) using log binomial regression analysis.

**Results:**

A total of 10,731 women reported cannabis use in pregnancy. Prevalence increased from 1.2% in 2012 to 1.8% in 2017 (*p*-trend, < 0.001), equivalent to a relative increase of 61% (relative risk [RR] 1.61, 95% confidence interval [CI] 1.51 to 1.72). The crude prevalence of cannabis use in pregnancy among women aged 15 to 24 years and in the lowest two area-level income quintiles was 6.7%, compared to 0.3% among women aged 35 years and over in the highest three income quintiles (RR 24.59, 95% CI 21.98 to 27.52). A majority (52.0%) of cannabis users were aged 15–24 years and 54.7% of users were in the lowest two income quintiles.

**Conclusion:**

Cannabis use in pregnancy has increased since 2012 in Ontario and was reported in about 2% of pregnancies in 2017. Increases were predominately among women of younger ages and those of lower SES, and these groups account for half of users. Promoting cannabis cessation in pregnancy could lead to improved perinatal and later childhood outcomes and reduce health inequalities.

**Electronic supplementary material:**

The online version of this article (10.17269/s41997-018-0148-0) contains supplementary material, which is available to authorized users.

## Résumé

**Objectif:**

Des modifications législatives prochaines légaliseront le cannabis et en généraliseront la disponibilité au Canada. Nous avons analysé le registre des naissances de l’Ontario pour en dégager les tendances récentes et les corrélats de la consommation de cannabis durant la grossesse.

**Méthode:**

Nous avons mené une étude de cohorte populationnelle rétrospective à partir des données du Registre et Réseau des Bons Résultats dès la naissance (BORN) de l’Ontario, qui couvre les naissances d’enfants vivants et les mortinaissances enregistrées entre avril 2012 et décembre 2017 en Ontario. Nous avons analysé les tendances de la consommation autodéclarée de cannabis durant la grossesse selon l’âge maternel et le statut socioéconomique régional par régression log-binomiale.

**Résultats:**

En tout, 10,731 femmes ont dit avoir consommé du cannabis durant la grossesse. La prévalence est passée de 1,2 % en 2012 à 1,8 % en 2017 (tendance *p* < 0,001), ce qui représente une hausse relative de 61 % (risque relatif [RR] 1,61, intervalle de confiance de 95% [IC] 1,51 à 1,72). La prévalence brute de la consommation de cannabis durant la grossesse chez les femmes de 15 à 24 ans dans les deux quintiles de revenu régionaux inférieurs était de 6,7 %, contre 0,3 % chez les femmes de 35 ans et plus dans les trois quintiles de revenu supérieurs (RR 24,59, IC de 95% 21,98 à 27,52). La majorité (52,0 %) des consommatrices de cannabis avait entre 15 et 24 ans, et 54,7 % des consommatrices appartenaient aux deux quintiles de revenu inférieurs.

**Conclusion:**

La consommation de cannabis durant la grossesse a augmenté depuis 2012 en Ontario et a été déclarée dans environ 2 % des grossesses en 2017. Les augmentations ont été constatées principalement chez les jeunes femmes et les femmes de faible statut socioéconomique; ces groupes représentent la moitié des consommatrices. Promouvoir l’arrêt du cannabis durant la grossesse pourrait donc améliorer les résultats périnatals et au cours de l’enfance et réduire les inégalités de santé.

## Introduction

In 2017, the Government of Canada introduced legislation to allow the consumption, sale, and distribution of cannabis (marijuana) for non-medical uses by October 2018 (Government of Canada 2016). Cannabis is the most widely used illicit drug in Canada, and the prevalence has been increasing in recent years following a period of decline from 2004 to 2011 (Government of Canada 2015). The 2015 Canadian Tobacco, Alcohol and Drugs Survey indicated that the prevalence of past-year cannabis use was 12% of the overall population (3.6 million individuals), but was 21% among young women and men 15 to 19 years of age (Government of Canada 2015). The prevalence among women increased from 6% in 2011 to 10% in 2015, although only 3% of women reported a weekly or greater frequency of use. Legalization of recreational cannabis in the American states of Washington and Colorado led to reductions in perceived harmfulness of cannabis and increases in use among 8th and 10th graders (Cerda et al. 2017). In Canada, legalization may likely increase the availability of cannabis (Pacula et al. 2013), decrease the perceptions of harm (Ko et al. 2015), and lead to further increases in use, including among pregnant women.

A 2017 review concluded that although robust evidence exists for certain therapeutic effects of cannabis, including treatment of chronic pain and as an antiemetic for chemotherapy-induced nausea, there exists only limited or moderate evidence for other purported therapeutic uses, including treatment of appetite and weight loss associated with HIV/AIDS, improving anxiety symptoms, and improving PTSD (National Academies of Sciences Engineering and Medicine- Health and Medicine Division; Board on Population Health and Public Health- Practice Committee on the Health Effects of Marijuana 2017). Acute effects of cannabis use include disruption of psychomotor function, global cognitive impairments, and impairments in abstract thinking and executive function (Crean et al. 2011; Hoch et al. 2015). Epidemiological research suggests that chronic cannabis use may lead to substance dependence in up to one in ten users (Hoch et al. 2015), and may have impacts on psychosocial development, educational attainment, and mental health in adolescents (Hall and Degenhardt 2009). Other effects on respiratory function and cardiovascular events have been suggested, although evidence remains limited (National Academies of Sciences Engineering and Medicine- Health and Medicine Division; Board on Population Health and Public Health- Practice Committee on the Health Effects of Marijuana 2017; Goyal et al. 2017). Cannabis intoxication, however, has been associated with a moderate and statistically significant increase in motor vehicle crash risk in an analysis of 28 observational studies (pooled odds ratio [OR] 1.36, 95% CI 1.15 to 1.61) (Rogeberg and Elvik 2016).

Potential health effects of perinatal cannabis exposure on pregnancy and neonatal outcomes have been investigated using various observational designs with inconsistent findings (Varner et al. 2014; Saurel-Cubizolles et al. 2014; Leemaqz et al. 2016; Hayatbakhsh et al. 2012; Fergusson et al. 2002; Conner et al. 2016; Gunn et al. 2016; El Marroun et al. 2009). Although statistically significant associations have been reported between prenatal cannabis use and low birth weight (LBW, < 2500 g) and admission to a neonatal intensive care unit (Gunn et al. 2016), it has not been robustly associated with fetal development, LBW, small for gestational age, or preterm birth after adjusting for tobacco use and other confounding factors (Fergusson et al. 2002; Conner et al. 2016; Witter and Niebyl 1990). A large study of 26,654 singleton births between 2009 and 2014 in Southwestern Ontario reported an adjusted OR of 2.7 (95% CI 1.7 to 4.4) for the association between history of cannabis use and LBW and an adjusted OR of 1.7 (95% CI 1.1 to 2.5) for < 10th percentile birth weight adjusted for history of tobacco smoking (Campbell et al. 2018). Inconsistencies in findings and methodological differences across epidemiological studies of cannabis exposure underscore the urgency for additional high-quality population-based data on this topic.

The Better Outcomes Registry and Network (BORN) is a comprehensive birth registry in the province of Ontario and encompasses nearly 40% of all births in Canada (Dunn et al. 2011). It contains robust data on prenatal screening, pregnancy complications, intrapartum events, admission to neonatal intensive care, newborn screening, and data on maternal exposures, including substance use. In this analysis, we evaluated recent trends and correlates of cannabis use in pregnancy.

## Methods

Research ethics board approval for this study was obtained from the Ottawa Health Science Network Research Ethics Board and the Children’s Hospital of Eastern Ontario.

### Study population and data source

The BORN Ontario birth registry captures all births occurring in the province. The routine data collection includes information on maternal demographics and health behaviours, pre-existing health problems, obstetric complications, and birth outcomes. Data are collected from medical records, clinical forms, and patient interviews when a woman is admitted to hospital to give birth. An ongoing program of data quality checks and formal training sessions assures a high level of data quality (Dunn et al. 2011).

For this study, we conducted a retrospective cohort analysis of the most recent available data from the BORN information system comprising women who delivered a singleton live birth or stillbirth in an Ontario hospital between April 1, 2012 and December 31, 2017.

### Primary outcome

Maternal self-report of cannabis use in pregnancy was determined from the BORN database using a multi-select variable which collected information on substance use during pregnancy, including cannabis, cocaine, hallucinogens, opioids, and other substances.

### Covariates

Maternal age was derived from maternal birth date and date of delivery. Area-level income quintiles and residential area population classification were assigned from the Canadian census based on data aggregated to a women’s local dissemination area. To assign these data, maternal postal codes in the database were linked using the Postal Code Conversion File Plus (PCCF+) version 6, developed by Statistics Canada (Statistics Canada 2016). This program assigns women to their dissemination area using six-digit postal codes and uses probabilistic assignment in cases where areas overlap more than one postal code. Smoking, alcohol exposure, and pre-existing diabetes and hypertension were included as covariates.

### Statistical analyses

We conducted descriptive analyses, including the Cochran-Armitage test to evaluate trends in cannabis use over time in the overall sample and by age groups and area-level income quintiles. Log binomial regression was used to assess the effect of year, maternal age, and income on rates of cannabis use, and to test for interaction between year and maternal age, and year and income. Multivariable models included year, age, income, and population size. A chi-square test was used for assessing differences in age and income distribution separately in cannabis users and in non-users.

## Results

The initial study sample comprised 783,419 pregnancy records. In total, 50,601 (6.5%) records were excluded due to missing information on cannabis use, maternal age, or area-level income. Records with maternal age less than 15 or greater than 50 were excluded (*n* = 219), yielding a final sample size of 732,818 for the analysis. Excluded participants were older, more likely to report alcohol use in pregnancy, to have diabetes, and higher income (Supplemental Table).

Among women with singleton live births in the analysis, 64.1% (*n* = 469,725) were aged 25 through 34 years, 13.4% were less than 25 years, and 22.5% were greater than 35 years of age. A majority (69.7%) were from urban areas with greater than 100,000 inhabitants and 24.8% (*n* = 182,009) fell into the highest income quintile based on residential postal code. 1.5% (*n* = 10,731) of women used cannabis in pregnancy and 9.1% used cigarettes at the first prenatal visit (Table 1). The lowest two income quintiles accounted for more than half (55%) of the population of cannabis users compared to about 30% of the population of non-users and these proportions were similar throughout the study period (*p* = 0.90). Cannabis use was substantially higher among women who reported alcohol and tobacco use in pregnancy (12.0% and 10.3%, respectively).

**Table 1:** Characteristics of pregnant women and cannabis users in Ontario, Canada, 2012–2017

	All singleton live births and stillbirths		Cannabis use during pregnancy		Prevalence of cannabis use (95% CI)
Variable	*n* (%)		*n* (%)		
All women	732,818	100.0	10,731	100.00	1.46 (1.44 to 1.49)
Year					
2012/13	122,519	16.7	1527	14.2	1.24 (1.18 to 1.31)
2013/14	125,890	17.2	1604	15.0	1.27 (1.21 to 1.34)
2014/15	127,355	17.4	1790	16.7	1.41 (1.34 to 1.47)
2015/16	127,268	17.4	1892	17.6	1.49 (1.42 to 1.55)
2016/17	129,929	17.7	2175	20.3	1.67 (1.60 to 1.74)
2017/17	99,857	13.6	1743	16.2	1.75 (1.66 to 1.83)
Maternal age at delivery					
15 to 24	98,437	13.4	5580	52.0	5.49 (5.33 to 5.64)
25 to 29	203,358	27.8	2822	26.3	1.39 (1.34 to 1.44)
30 to 34	266,367	36.5	1622	15.1	0.61 (0.58 to 0.64)
35 and older	164,656	22.5	707	6.6	0.43 (0.40 to 0.46)
Area-level income quintile*					
Lowest	111,222	15.2	3460	32.2	3.11 (3.01 to 3.21)
Medium-low	113,894	15.5	2414	22.5	2.12 (2.04 to 2.20)
Middle	152,153	20.8	2121	19.8	1.39 (1.34 to 1.45)
Medium-high	173,540	23.7	1667	15.3	0.96 (0.91 to 1.01)
Highest	182,009	24.8	1069	10.0	0.58 (0.55 to 0.62)
Population size classification*					
Rural area	97,897	13.4	1829	17.0	1.87 (1.78 to 1.95)
1000 to 29,999 population	66,971	9.1	1510	14.1	2.25 (2.14 to 2.37)
30,000 to 99,999 population	57,542	7.9	1730	16.1	3.01 (2.87 to 3.15)
100,000 or greater population	510,408	69.7	5662	52.8	1.11 (1.08 to 1.14)
Alcohol exposure in pregnancy, *n* (%)					
None	708,291	96.7	8191	76.3	1.16 (1.13 to 1.18)
Any	16,095	2.2	1935	18.0	12.02 (11.52 to 12.52)
Amount unknown or missing	8432	1.2	605	5.6	7.18 (6.62 to 7.72)
Maternal smoking at time of labour/admission, *n* (%)					
None	659,654	90.0	4192	39.1	0.64 (0.62 to 0.65)
Any	54,807	7.5	5913	55.1	10.79 (10.53 to 11.05)
Amount unknown or missing	18,357	2.5	626	5.8	3.41 (3.15 to 3.67)
Maternal smoking at first prenatal visit, *n* (%)					
None	640,572	87.4	3187	29.7	0.50 (0.48 to 0.52)
Any	66,441	9.1	6855	63.9	10.32 (10.09 to 10.55)
Amount unknown or missing	25,805	3.5	689	6.4	2.67 (2.47 to 2.87)
Diabetes, *n* (%)					
None	659,332	89.9	9999	93.2	1.5 (1.48 to 1.55)
Any	52,419	7.2	435	4.1	0.83 (0.75 to 0.91)
Hypertensive disorder in pregnancy, *n* (%)					
None	692,229	94.5	10,095	94.1	1.46 (1.43 to 1.49)
Any	34,412	4.7	438	4.1	1.27 (1.15 to 1.39)

*Based on residential postal code

**Fig. 1:**
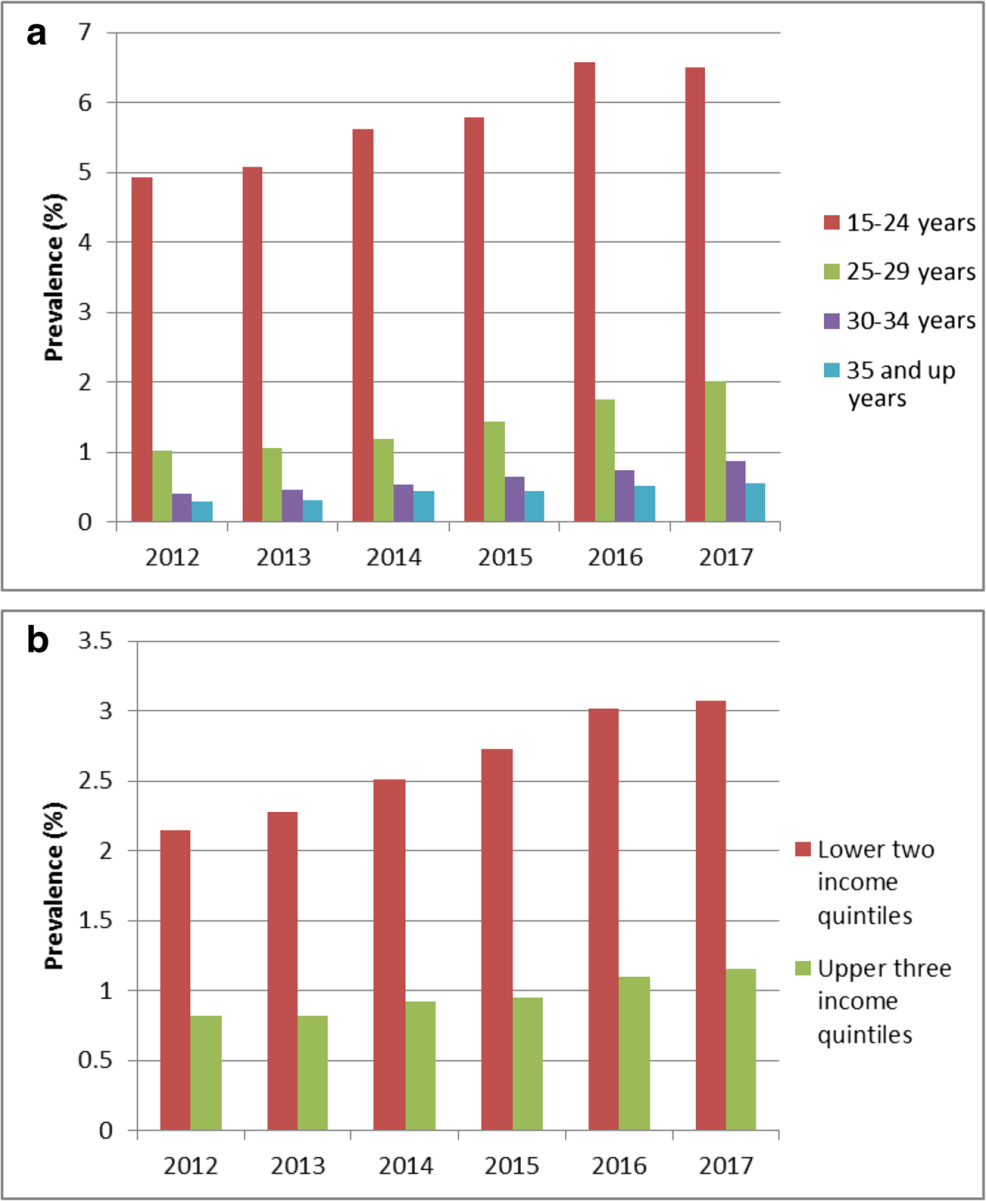
Self-reported cannabis use in pregnancy in Ontario, by (**a**) age (*p*-trend < 0.001 in four age groups) and (**b**) area-level income (*p*-trend < 0.001 in two income groups), 2012–2017

In Ontario, the overall prevalence of cannabis use in pregnancy increased from 1.2% in 2012 to 1.8% in 2017 (*p*-trend, < 0.001). This equates to a relative increase of 61% between 2012 and 2017 (relative risk [RR] 1.61, 95% confidence interval [CI] 1.51 to 1.72) adjusting for maternal age, income, and population size (Table 2). Prevalence of prenatal cannabis use was highest among women aged 15–24 years, and increased from 4.9% in 2012 to 6.5% in 2017 (Fig. 1a). A test of interaction indicated that increases over time differed by age group (*p* < 0.001). There was an 18% relative decrease in prevalence with each additional year of maternal age (RR 0.82, 95% CI 0.819–0.825). Cannabis use varied by income, with women in the lowest two quintiles reporting the highest usage (2.2% in 2012, increasing to 3.1% in 2017) as compared to women in the highest three quintiles (0.8% in 2012, and increasing to 1.2% in 2017) (Fig. 1b). A test of time by area-level income interaction indicated variability in trends across income level (*p* < 0.001). The relative risk for cannabis use in the lowest compared to the highest income quintile was 5.30 (95% CI 4.95 to 5.67), and attenuated to 3.23 (95% CI 3.02 to 3.46) after adjusting for maternal age, year, and population size.

**Table 2:** Relative risk of cannabis use by year, age, income, and population size among pregnant women in Ontario, 2012–2017

	Relative risk (95% CI)	Adjusted^a^ relative risk (95% CI)
Year^*^		
2012/13	1.0 (Ref.)	1.0 (Ref.)
2013/14	1.02 (0.95 to 1.10)	1.05 (0.98 to 1.13)
2014/15	1.13 (1.05 to 1.21)	1.18 (1.11 to 1.27)
2015/16	1.19 (1.12 to 1.28)	1.29 (1.21 to 1.38)
2016/17	1.34 (1.26 to 1.43)	1.51 (1.41 to 1.61)
2017/17	1.40 (1.31 to 1.50)	1.61 (1.51 to 1.72)
Maternal age at delivery^*,**^		
15 to 24	13.20 (12.21 to 14.27)	10.88 (10.05 to 11.77)
25 to 29	3.23 (2.98 to 3.51)	2.97 (2.73 to 3.22)
30 to 34	1.12 (1.30 to 1.55)	1.41 (1.29 to 1.53)
35 and older	1.0 (Ref.)	1.0 (Ref.)
Area-level income quintile^*^		
Lowest	5.30 (4.95 to 5.67)	3.23 (3.02 to 3.46)
Medium-low	3.61 (3.36 to 3.88)	2.23 (2.07 to 2.39)
Middle	2.37 (2.21 to 2.55)	1.65 (1.54 to 1.78)
Medium-high	1.64 (1.52 to 1.77)	1.30 (1.20 to 1.40)
Highest	1.0 (Ref.)	1.0 (Ref.)
Population size classification^*^		
Rural area	1.68 (1.60 to 1.77)	1.47 (1.40 to 1.55)
1000 to 29,999 population	2.03 (1.92 to 2.15)	1.56 (1.48 to 1.65)
30,000 to 99,999 population	2.71 (2.57 to 2.86)	1.89 (1.79 to 2.00)
100,000 or greater population	1.0 (Ref.)	1.0 (Ref.)

*p < 0.001 for trend, Cochran-Armitage test

**p < 0.001 age*year interaction

^a^Models for year, age, income, and population size include these four variables

A combined indicator of age and area-level income indicated that, compared to those aged 35 years and above in the highest three quintiles for income, those aged 15–24 years were significantly more likely to use cannabis in pregnancy in the bottom two quintiles (RR 24.59, 95% CI 21.98 to 27.52) and upper three quintiles (RR 17.55, 95% CI 15.66 to 19.76) of area-level income (Table 3). Across all ages, the relative risk of cannabis use in pregnancy was higher among women in the bottom two quartiles of area-level income.

**Table 3:** Association of categories of age and area-level income on cannabis use in pregnancy, Ontario, Canada, 2012–2017, *n* = 732,818

	Bottom two income quintiles, *n* = 225,116				Upper three income quintiles, *n* = 507,702			
Cannabis use	15 to 24 y, *n* = 46,905	25 to 29 y, *n* = 68,881	30 to 34 y, *n* = 67,849	35 y and over, *n* = 41,481	15 to 24 y, *n* = 51,532	25 to 29 y, *n* = 134,477	30 to 34 y, *n* = 198,518	35 y and over, *n* = 123,175
Reported use, *n* (%) [95% CI]	3128 (6.7) [6.4–6.9]	1552 (2.3) [2.1–2.4]	821 (1.2) [1.1–1.3]	373 (0.9) [0.8–1.0]	2452 (4.8) [4.6–4.9]	1270 (0.9) [0.9–1.0]	801 (0.4) [0.38–0.43]	334 (0.3) [0.24–0.30]
Rate ratio (95% CI)	24.59 (21.98 to 27.52)	8.31 (7.39 to 9.35)	4.46 (3.93 to 5.07)	3.32 (2.86 to 3.84)	17.55 (15.66 to 19.67)	3.48 (3.09 to 3.93)	1.49 (1.31 to 1.69)	1.0 (Ref.)

Among those who reported cannabis use in pregnancy, the majority (52.0%) were aged 15–25 years, although due to increases in overall usage, this proportion declined with time from 60.1% in 2012 to 42.7% in 2017 (Fig. S1). There was a corresponding increase in the proportion of users at older ages over time between 2012 and 2017 (*p* < 0.001).

## Discussion

There has been an increase in cannabis use among pregnant women in Ontario since 2012, and the increase occurred predominately among younger women and those of lower socio-economic status (SES). In these groups, the prevalence of self-reported cannabis use during pregnancy was 5.5% and 3.1%, respectively, compared with 1.8% in the overall obstetrical population. Young women under 25 years of age and women from lower area-level SES represented more than half of all women who reported using cannabis during pregnancy, suggesting that the burden of potential health implications of cannabis use in pregnancy may be greater among these women.

Previous studies have indicated an association between prenatal cannabis use and adverse pregnancy outcomes such as preterm birth, small for gestational age, and admission to neonatal intensive care (Leemaqz et al. 2016; Hayatbakhsh et al. 2012; Campbell et al. 2018). In addition, there is evidence to suggest an increased risk of adverse developmental consequences in children with intrauterine exposure to cannabis (Fried and Watkinson 1990), although few studies exist with adequate follow-up (Hayes et al. 1991; McLemore and Richardson 2016). With forthcoming legalization leading to increased availability of cannabis in Canada, it is likely that the prevalence of prenatal exposure will continue to increase. Identification of women who use cannabis in pregnancy may facilitate interventions to improve obstetrical and neonatal outcomes (Fried and Makin 1987). The potential for underreporting of cannabis exposure in pregnancy is a challenge for understanding prevalence and identifying opportunities for intervention (Cook et al. 2017). BORN data on the use of cannabis in pregnancy are from self-reports, which are likely to be influenced by stigma, social desirability bias, and fear of intervention by child protection services (Greaves and Poole 2004; Stone 2015; Yonkers et al. 2011; Jacobson et al. 1991; Johnson and Fendrich 2005). Previous studies have attempted to validate self-reports of cannabis with urinary biomarkers of exposures and have found moderate correlation (Markovic et al. 2000; El Marroun et al. 2011). The authors of the Generation R study in Rotterdam suggested that despite the challenges in obtaining accurate information on cannabis use in pregnancy, self-report does seem to be a reliable method for determining use during pregnancy in epidemiological studies (El Marroun et al. 2009). Although underreporting may affect prevalence estimates for cannabis exposure, our associations between maternal age and SES and cannabis use may also be influenced if underlying biases in self-reports are patterned by age and/or SES. In addition, women with missing data on cannabis exposure were more likely to be older and in higher quintiles of area-level income. If this patterning of missing data was correlated with cannabis use, our associations between these characteristics and cannabis use may be overestimated. However, only 5% of the cohort was missing cannabis exposure information and it is unlikely that a potential correlation would substantially alter any of the reported effects.

The BORN data closely align with data from Canada and the United States. A recent study from Ontario indicated that pregnant women were more likely to report cannabis use if they lived below the low-income cutoff and were single mothers (Seabrook et al. 2018). Data from the US indicate that cannabis use among both pregnant and non-pregnant women of reproductive age has increased between 2002 and 2015 (Brown et al. 2017; Volkow et al. 2017). Among 200,510 women aged 18–44 who responded to the US National Survey on Drug Use and Health from 2002 to 2014, the adjusted prevalence of last-month cannabis use for pregnant women increased from 2.4% in 2002 to 3.9% in 2014, with an adjusted prevalence of 7.5% among those aged 18–25 (Brown et al. 2017). For non-pregnant women, a similar increasing trend was observed, from 6.3% last-month use in 2002, to 9.3% in 2014 (Brown et al. 2017). Across the literature, the prevalence of self-reported cannabis use during pregnancy varies between 1% and 6%, and may vary by trimester (Finnegan 2013).

Previous research has suggested that cannabis use in pregnancy is associated with a low perception of risk among mothers and this may be reflective of societal beliefs, including those of healthcare providers (Ko et al. 2015). Qualitative data have indicated that in prenatal visits among women who disclosed cannabis use to their obstetrical provider, nearly half of providers did not respond to the disclosure or offer counseling (Holland et al. 2016). Proposed changes to legislation and other societal trends which normalize the usage of cannabis could lead to further increases in the prevalence of prenatal usage in Canada and this may cause an increase in the burden of adverse pregnancy outcomes, especially among younger women or those of lower SES.

With 10,731 women who self-reported cannabis use in pregnancy, this is one of the largest studies of this topic, due to its being based on one of the largest perinatal databases globally. This study has identified that cannabis use disproportionately affects women from more vulnerable segments of society. From these data and given forthcoming legal changes to cannabis use, increases in prenatal screening for cannabis use may be warranted (El Marroun et al. 2011), in order to accurately identify women and neonates for intervention. Open communication between health practitioners and pregnant women to promote cannabis cessation in pregnancy may improve perinatal and potentially later childhood outcomes and reduce health inequalities.

## Electronic supplementary material

ESM 1 (DOCX 17 kb)

ESM 2 (DOCX 110 kb)

## References

[CR1] Brown QL, Sarvet AL, Shmulewitz D, Martins SS, Wall MM, Hasin DS (2017). Trends in marijuana use among pregnant and nonpregnant reproductive-aged women, 2002-2014. JAMA.

[CR2] Campbell EE, Gilliland J, Dworatzek PDN, De Vrijer B, Penava D, Seabrook JA (2018). Socioeconomic Status and Adverse birth outcomes: a population-based Canadian sample. Journal of Biosocial Science.

[CR3] Cerda M, Wall M, Feng T, Keyes KM, Sarvet A, Schulenberg J (2017). Association of state recreational marijuana laws with adolescent marijuana use. JAMA Pediatrics.

[CR4] Conner SN, Bedell V, Lipsey K, Macones GA, Cahill AG, Tuuli MG (2016). Maternal marijuana use and adverse neonatal outcomes: a systematic review and meta-analysis. Obstetrics and Gynecology.

[CR5] Cook JL, Green CR, de la Ronde S, Dell CA, Graves L, Morgan L (2017). Screening and management of substance use in pregnancy: a review. Journal of Obstetrics and Gynaecology Canada.

[CR6] Crean RD, Crane NA, Mason BJ (2011). An evidence based review of acute and long-term effects of cannabis use on executive cognitive functions. Journal of Addiction Medicine.

[CR7] Dunn S, Bottomley J, Ali A, Walker M (2011). 2008 Niday Perinatal Database quality audit: report of a quality assurance project. Chronic Diseases and Injuries in Canada.

[CR8] El Marroun H, Tiemeier H, Steegers EAP, Jaddoe VWV, Hofman A, Verhulst FC (2009). Intrauterine cannabis exposure affects fetal growth trajectories: the generation R study. Journal of the American Academy of Child and Adolescent Psychiatry.

[CR9] El Marroun H, Tiemeier H, Jaddoe V, Hofman A, Verhulst F, van den Brink W (2011). Agreement between maternal cannabis use during pregnancy according to self-report and urinalysis in a population-based cohort: the Generation R Study. European Addiction Research.

[CR10] Fergusson DM, Horwood LJ, Northstone K, Team AS (2002). Maternal use of cannabis and pregnancy outcome. BJOG: An International Journal of Obstetrics & Gynaecology.

[CR11] Finnegan L (2013). Substance abuse in Canada: licit and illicit drug use during pregnancy: maternal, neonatal and early childhood consequences.

[CR12] Fried PA, Makin JE (1987). Neonatal behavioural correlates of prenatal exposure to marihuana, cigarettes and alcohol in a low risk population. Neurotoxicology and Teratology.

[CR13] Fried PA, Watkinson B (1990). 36- and 48-month neurobehavioral follow-up of children prenatally exposed to marijuana, cigarettes, and alcohol. Journal of Developmental and Behavioral Pediatrics.

[CR14] Government of Canada (2015). Canadian Tobacco Alcohol and Drugs Survey (CTADS). https://www.canada.ca/en/health-canada/services/canadian-tobacco-alcohol-drugs-survey/2015-summary.html.

[CR15] Government of Canada (2016). A framework for the legalization and reguation of cannabis in Canada.

[CR16] Goyal H, Awad HH, Ghali JK (2017). Role of cannabis in cardiovascular disorders. Journal of Thoracic Disease.

[CR17] Greaves L., Poole N. (2004) Victimized or validated? Responses to substance-using pregnant women. *Canadian Woman Studies*, *24*(1), 87–92.

[CR18] Gunn JKL, Rosales CB, Center KE, Nuñez A, Gibson SJ, Christ C (2016). Prenatal exposure to cannabis and maternal and child health outcomes: a systematic review and meta-analysis. BMJ Open.

[CR19] Hall W, Degenhardt L (2009). Adverse health effects of non-medical cannabis use. Lancet.

[CR20] Hayatbakhsh MR, Flenady VJ, Gibbons KS, Kingsbury AM, Hurrion E, Mamun AA (2012). Birth outcomes associated with cannabis use before and during pregnancy. Pediatric Research.

[CR21] Hayes JS, Lampart R, Dreher MC, Morgan L (1991). Five-year follow-up of rural Jamaican children whose mothers used marijuana during pregnancy. The West Indian Medical Journal.

[CR22] Hoch E, Bonnetn U, Thomasius R, Ganzer F, Havemann-Reinecke U, Preuss UW (2015). Risks associated with the non-medicinal use of cannabis. Deutsches Ärzteblatt International.

[CR23] Holland CL, Rubio D, Rodriguez KL, Kraemer KL, Day N, Arnold RM (2016). Obstetric health care providers’ counseling responses to pregnant patient disclosures of marijuana use. Obstetrics and Gynecology.

[CR24] Jacobson SW, Jacobson JL, Sokol RJ, Martier SS, Ager JW, Kaplan MG (1991). Maternal recall of alcohol, cocaine, and marijuana use during pregnancy. Neurotoxicology and Teratology.

[CR25] Johnson T, Fendrich M (2005). Modeling sources of self-report bias in a survey of drug use epidemiology. Annals of Epidemiology.

[CR26] Ko JY, Farr SL, Tong VT, Creanga AA, Callaghan WM (2015). Prevalence and patterns of marijuana use among pregnant and nonpregnant women of reproductive age. American Journal of Obstetrics and Gynecology.

[CR27] Leemaqz SY, Dekker GA, McCowan LM, Kenny LC, Myers JE, Simpson NAB (2016). Maternal marijuana use has independent effects on risk for spontaneous preterm birth but not other common late pregnancy complications. Reproductive Toxicology.

[CR28] Markovic N, Ness RB, Cefilli D, Grisso JA, Stahmer S, Shaw LM (2000). Substance use measures among women in early pregnancy. American Journal of Obstetrics and Gynecology.

[CR29] McLemore GL, Richardson KA (2016). Data from three prospective longitudinal human cohorts of prenatal marijuana exposure and offspring outcomes from the fetal period through young adulthood. Data in Brief.

[CR30] National Academies of Sciences Engineering and Medicine- Health and Medicine Division; Board on Population Health and Public Health- Practice Committee on the Health Effects of Marijuana. (2017). The health effects of cannabis and cannabinoids: the current state of evidence and recommendations for research. The National Academies Collection: Reports funded by National Institutes of Health. Washington (DC).

[CR31] Pacula, R. L., Powell D., Heaton P., Sevigny E. L. (2015) Assessing the effects of medical marijuana laws on marijuana and alcohol use: the devil is in the details*.**J Policy Anal Manage, 34*(1), 7–31*.* 10.1002/pam.21804 PMC4315233 25558490

[CR32] Rogeberg O, Elvik R (2016). The effects of cannabis intoxication on motor vehicle collision revisited and revised. Addiction.

[CR33] Saurel-Cubizolles MJ, Prunet C, Blondel B (2014). Cannabis use during pregnancy in France in 2010. BJOG: An International Journal of Obstetrics & Gynaecology.

[CR34] Seabrook JA, Woods N, Clark A, de Vrijer B, Penava D, Gilliland J (2018). The association between alcohol outlet accessibility and adverse birth outcomes: a retrospective cohort study. Journal of Neonatal-Perinatal Medicine.

[CR35] Statistics Canada (2016). Postal Code Conversion File Plus (PCCF+) version 6D.

[CR36] Stone R (2015). Pregnant women and substance use: fear, stigma, and barriers to care. Health & Justice.

[CR37] Varner MW, Silver RM, Hogue CJR, Willinger M, Parker CB, Thorsten VR (2014). Association between stillbirth and illicit drug use and smoking during pregnancy. Obstetrics and Gynecology.

[CR38] Volkow ND, Han B, Compton WM, Blanco C (2017). Marijuana use during stages of pregnancy in the United States. Annals of Internal Medicine.

[CR39] Witter FR, Niebyl JR (1990). Marijuana use in pregnancy and pregnancy outcome. American Journal of Perinatology.

[CR40] Yonkers KA, Howell HB, Gotman N, Rounsaville BJ (2011). Self-report of illicit substance use versus urine toxicology results from at-risk pregnant women. Journal of Substance Use.

