# Supplementary material for: Trends and correlates of cannabis use in pregnancy: a population-based study in Ontario, Canada from 2012 to 2017
Source: Can J Public Health. 2018 Nov 1;110(1):76–84. doi: 10.17269/s41997-018-0148-0 (PMC6335373; doi:10.17269/s41997-018-0148-0)
Supplement: Supplementary file 1 — (DOCX 17 kb) [file 41997_2018_148_MOESM1_ESM.docx]

**SUPPLEMENTAL APPENDIX**

**Figure S1**: Age distribution of women reporting cannabis use in pregnancy in Ontario, 2012 to 2017
