# Supplementary material for: Trends and correlates of cannabis use in pregnancy: a population-based study in Ontario, Canada from 2012 to 2017
Source: Can J Public Health. 2018 Nov 1;110(1):76–84. doi: 10.17269/s41997-018-0148-0 (PMC6335373; doi:10.17269/s41997-018-0148-0)
Supplement: Supplementary file 2 — (DOCX 110 kb) [file 41997_2018_148_MOESM2_ESM.docx]

**Supplemental Table**: Characteristics of pregnant women with complete and missing cannabis information in Ontario, 2012-2016, n=783,419*

| **Variable** | | | **Complete cannabis information, n=740,653** | | **Missing cannabis information, n=42,766** | |
| --- | --- | --- | --- | --- | --- | --- |
| Maternal age at delivery** | | | n | % | n | % |
|  | 15 to 24 | | 100311 | 13.55 | 3715 | 8.69 |
|  | 25 to 29 | | 205402 | 27.74 | 8141 | 19.04 |
|  | 30 to 34 | | 268497 | 36.26 | 16428 | 38.42 |
|  | 35 and older | | 166036 | 22.42 | 14449 | 33.79 |
| Income quintile** | | |  |  |  |  |
|  | Lowest | | 111305 | 15.18 | 5252 | 12.41 |
|  | Medium-low | | 113970 | 15.54 | 5246 | 12.40 |
|  | Middle | | 152247 | 20.76 | 7217 | 17.06 |
|  | Medium-high | | 173654 | 23.68 | 9352 | 22.10 |
|  | Highest | | 182134 | 24.84 | 15243 | 36.03 |
| Population size classification** | | |  |  |  |  |
|  | Rural area | | 100208 | 13.59 | 2142 | 5.02 |
|  | 1,000 to 29,000 population | | 67327 | 9.13 | 1308 | 3.07 |
|  | 30,000 to 99,999 population | | 57653 | 7.82 | 1892 | 4.44 |
|  | 100,000 or greater population | | 511972 | 69.45 | 37309 | 87.48 |
| Alcohol Exposure in Pregnancy** | | |  |  |  |  |
|  | None | | 715673 | 97.76 | 5763 | 95.49 |
|  | Any | | 16398 | 2.24 | 272 | 4.51 |
| Maternal smoking at time of labour/admission** | | |  |  |  |  |
|  | None | | 666079 | 92.25 | 20963 | 93.59 |
|  | Any | | 55920 | 7.75 | 1435 | 6.41 |
| Maternal smoking at first prenatal visit** | | |  |  |  |  |
|  | | None | 646742 | 90.51 | 17361 | 92.39 |
|  | | Any | 67809 | 9.49 | 1431 | 7.61 |
| Any diabetes** | | | 53043 | 7.37 | 2662 | 11.85 |
| Any hypertensive disorder in pregnancy | | | 34828 | 4.74 | 1064 | 4.87 |

*Totals do not equal this number due to missing information on other variables

**Chi-square p-value <0.001
